# *Notes from the Field:* Outbreak of *Salmonella* Enteritidis at a Correctional Facility Using Mechanically Separated Chicken — Nebraska, 2022

**DOI:** 10.15585/mmwr.mm7128a4

**Published:** 2022-07-15

**Authors:** Sadie J. Oppegard, Andrea R. Bethke, Benjamin A. Davy, Ashley E. Johnson, Justin L. Daniel, Scott E. Holmes

**Affiliations:** ^1^Nebraska Department of Health and Human Services; ^2^Lincoln-Lancaster County Health Department, Lincoln, Nebraska.

On January 14, 2022, the Lincoln-Lancaster County (Nebraska) Health Department (LLCHD) notified the Nebraska Department of Health and Human Services (NDHHS) of two cases of laboratory-confirmed *Salmonella* in inmates at a correctional facility (facility A). LLCHD initiated an investigation in collaboration with NDHHS to identify the source of the outbreak and develop recommendations. The investigation linked consumption of mechanically separated chicken to illness. Mechanically separated chicken, which is produced at chicken processing facilities by separating edible chicken from bone and cartilage under pressure, is frequently purchased for use in institutions, such as prisons, jails, and correctional facilities because of its affordability (*1*,*2*).

Staff members at facility A reported approximately 100 inmates experienced gastrointestinal symptoms during a period of a few days; no staff member reported illness. LLCHD conducted open-ended interviews with ill inmates. Because the facility was experiencing a concurrent outbreak of COVID-19, and access to inmates for interviews was limited, it is likely that additional cases existed among noninterviewed and untested inmates beyond the total cases identified in the investigation. Inmates who were designated food handlers were prioritized for interviews because of transmission risk to others; untested inmates were able to seek care through facility A medical staff. A probable case was defined as the onset of diarrhea, stomach cramps, or vomiting during January 9–11, 2022, but without a positive stool culture, in an inmate at facility A; a confirmed case was defined as isolation of *Salmonella* serotype Enteritidis highly related to the outbreak strain (within three alleles) by core genome multilocus sequence typing in a clinical specimen. LLCHD conducted an environmental assessment on January 15, 2022. A list of food handlers, food menus for January, and temperature logs were requested. During the environmental assessment, a sample of raw, unopened mechanically separated chicken from a 50-lb intact box from the same shipment used to prepare a meal on January 8, 2022, was collected for testing.

A total of 15 cases of *S*. Enteritidis infection were identified, including five confirmed and 10 probable cases. The median patient age was 39 years (range = 24–62 years); 93% were male and two patients were hospitalized. All 15 cases occurred in food workers, all of whom reported eating chili that had been prepared from the raw mechanically separated chicken product.

*S.* Enteritidis that genetically matched the outbreak strain was isolated from the raw mechanically separated chicken sample. The Food Safety and Inspection Service agency of the U. S. Department of Agriculture (USDA) was notified by NDHHS of the poultry product matching the outbreak. However, *Salmonella* spp. are not considered adulterants of raw poultry products because *Salmonella* is regularly present on poultry products, and safe standard cooking practices typically destroy *Salmonella* bacteria; therefore, no regulatory action was taken* (*3*).

The environmental assessment identified potential food safety risks in both incomplete thawing and cooking processes for mechanically separated chicken. Qualitative interviews revealed that the mechanically separated chicken product was sometimes still frozen or partially frozen at the time of cooking; this process was also observed by LLCHD on a follow-up site visit. Cooking temperatures were not routinely monitored while food was being prepared, and LLCHD was unable to verify that the mechanically separated chicken product reached a safe internal cooking temperature before being served.

LLCHD provided recommendations for prevention of foodborne outbreaks to facility A, which included policies for excluding ill workers from food preparation, increased thawing time for mechanically separated chicken under refrigeration, routine monitoring and recording of cooking temperatures, and adjustment of meals to smaller preparation volumes to mitigate food safety risks. LLCHD worked with facility management to implement new policies and procedures for food safety practices.

This outbreak of *S.* Enteritidis was associated with mechanically separated chicken and substandard cooking processes. Mechanically separated chicken products routinely tested by USDA have indicated a higher prevalence of *Salmonella* spp. (82.9%) than ground chicken (39.0%) and other comminuted chicken products (41.7%) sampled and tested during June 1, 2013–December 31, 2014 (*4*). Mechanically separated chicken is typically used as an ingredient in other processed meat products, such as hot dogs, which can be thermally processed to ensure they are cooked to a safe internal temperature (*1*). Several previous state and multistate salmonellosis outbreaks have implicated mechanically separated chicken as the source of infection in correctional facilities^†^ (*1*). Populations who are obligated to eat in certain locations and who have limited choice regarding what they eat are dependent on societal responsibility to ensure their health and the facility’s food safety procedures (*5*). Mitigating the risks of food handling and processing and cooking in vulnerable populations and institutions requires two key actions: 1) providing a less highly contaminated poultry product in the absence of contamination threshold regulatory requirements for poultry products and 2) implementing a preventive food safety management system to ensure thawing, cooking, and cooling processes meet food safety requirements.

## Footnotes

*
https://www.fsis.usda.gov/sites/default/files/media_file/2021-09/FSIS-GD-2013-0003.pdf

†
https://www.cdc.gov/salmonella/heidelberg-01-14/index.html

## References

[1] Hutchinson JA, Wheeler C, Mohle-Boetani JC. Outbreak epidemiologically linked with a composite product of beef, mechanically separated chicken and textured vegetable protein contaminated with multiple serotypes of *Salmonella enterica* including multidrug-resistant Infantis, California 2016. Epidemiol Infect 2018;146:430–6. 10.1017/S0950268817002941 29307318 PMC9134547

[2] Ozkececi RB, Karakaya M, Yilmaz MT, Saricoban C, Ockerman HW. The effect of carcass part and packaging method on the storage stability of mechanically deboned chicken meat. J Muscle Foods 2008;19:288–301. 10.1111/j.1745-4573.2008.00118.x

[3] US Government Accountability Office. Food safety: USDA should take further action to reduce pathogens in meat and poultry products. Washington, DC: US Government Accountability Office; 2019. Accessed July 8, 2022. https://www.gao.gov/assets/gao-18-272.pdf

[4] Food Safety and Inspection Service. Progress report on *Salmonella* and *Campylobacter* testing of raw meat and poultry products,1998–2014. Washington, DC: US Department of Agriculture, Food Safety and Inspection Service; 2016. https://www.fsis.usda.gov/sites/default/files/media_file/2021-02/Progress-Report-Salmonella-Campylobacter-CY2014.pdf

[5] Marlow MA, Luna-Gierke RE, Griffin PM, Vieira AR. Foodborne disease outbreaks in correctional institutions—United States, 1998–2014. Am J Public Health 2017;107:1150–6. 10.2105/AJPH.2017.303816 28520482 PMC5463225

